# Immediate Effects of Aquatic Therapy on Balance in Older Adults with Upper Limb Dysfunction: An Exploratory Study

**DOI:** 10.3390/ijerph17249434

**Published:** 2020-12-16

**Authors:** Maria Graça, José Alvarelhão, Rui Costa, Ricardo J. Fernandes, Andrea Ribeiro, Daniel Daly, João Paulo Vilas-Boas

**Affiliations:** 1 Research Centre for Physical Activity, Health and Leisure, Faculty of Sport, University of Porto, 4200-450 Porto, Portugal; 2 School of Health Sciences, University of Aveiro, 3810-193 Aveiro, Portugal; jalvarelhao@ua.pt (J.A.); rcosta@ua.pt (R.C.); 3 Porto Biomechanics Laboratory (LABIOMEP-UP), University of Porto, 4200-450 Porto, Portugal; ricfer@fade.up.pt (R.J.F.); andrear@ufp.edu.pt (A.R.); jpvb@fade.up.pt (J.P.V.-B.); 4 Centre of Research, Education, Innovation and Intervention in Sport, Faculty of Sport, University of Porto, 4200-450 Porto, Portugal; 5 School of Health, Fernando Pessoa University, 4200-253 Porto, Portugal; 6 Department of Movement Sciences, KU Leuven, 3001 Leuven, Belgium; daniel.daly@kuleuven.be

**Keywords:** single aquatic intervention, outcomes, functional performance

## Abstract

Background: Aquatic physiotherapy has been shown to be effective in developing balance, strength, and functional reach over time. When dealing with immediate effects, the literature has concentrated more on the body’s physiological response to the physical and mechanical properties of water during passive immersion. The purpose of this study was to evaluate the effects of a single 45-min active aquatic physiotherapy session on standing balance and strength, and its relationship with functional reach in persons 55 years and older with upper limb dysfunction. Methods: The intervention group (*n* = 12) was assessed before and after a single aquatic physiotherapy session, while the control group (*n* = 10) was evaluated before and after 45 min of sitting rest. Functional assessment was made using the visual analogue pain scale (points), step test (repetitions), functional reach test (cm), and global balance-standing test on a force platform (% time). A two-way repeated-measures ANOVA was applied (*p* < 0.05). Results: The intervention group showed non-significant improvements between measurement before and after the intervention: Pain: 6.2 ± 1.9 vs. 5.2 ± 2.3 cm, steps: 7.0 ± 2.0 vs. 7.4 ± 1.8 repetitions, reach: 9.1 ± 2.8 vs. 10.4 ± 3.8 cm, and balance: 61.7 ± 5.9 vs. 71.3 ± 18.2% time in balance on the platform. The control group showed fewer changes but had better baseline values. A comparison between groups with time showed no significant differences in these changes. Conclusions: No significant immediate effects were found for one session of aquatic physiotherapy applied to patients older than 55 years with upper limb dysfunction.

## 1. Introduction

Recent research has studied the benefits of exercise on the enhancement of functional capacity and reduction in risk and rate of falling in older adults [1]. Exercise interventions should focus on increasing the muscle strength, muscle mass, improving balance, and increasing gait ability [2,3]. Exercise programs that stimulate several physical capacities, such as muscle strength and endurance, cardiorespiratory fitness, balance and coordination, appear to result in greater improvements in aging adults’ ability to perform activities of daily living [3,4,5].

Aquatic therapy has been proposed as an effective therapeutic approach to maintain performance, improve balance, and reduce the risk of falls in older adults over the long-term [6,7]. It has several advantages compared to non-aquatic exercise, due to the physical properties of water [8,9,10]. Buoyancy acts on the body to reduce the vertical load on the joints [11] and this antigravity effect may reduce the perception of fatigue and aid energy conservation [12]. Furthermore, the viscosity of water and the associated resistive hydrodynamic force requires the individual to exert more force when performing immersed movements [11]. In other words, aquatic therapy allows high-intensity exercise, while ensuring both low joint impact and greater comfort for the individual.

The thermal properties of water afford a higher capacity to dissipate heat, which helps maintain a constant body temperature and thus better controlling oedema and inflammation, diminishing fatigue and pain, and promoting recovery in one single immersion [13]. Another important property of water is hydrostatic pressure, which leads to the improved conduction of fluids from the extremities towards the central cavity of the human body [13]. The decrease in perception of fatigue may also be due to reduced neuromuscular responses during water immersion [12,14]. Other advantages include low risk of injury from falling and the consequent lack of fear of falling during water exercise with a moderate and high intensity load [15,16].

Although several studies show the efficacy of aquatic therapy on balance gain, pain relief, and functionality in longer term interventions for older adults [17,18], the sustainable effects [19] or the immediate effects, e.g., of a single aquatic therapy session remain unclear for functional tests. In a study of immediate effects, Waller et al. (2017) looked at the walking speed in persons with mild knee osteoarthritis after a 4-month intervention. They found increased walking speed and decreased fat mass. However, in a 12-month follow up, fat mass returned to the base line while walking speed maintained its improvement. These authors did not examine at what point in the 4-month intervention (3× week) the actual improvements were reached. They also found regression in outcome measures with the exception of walking speed in a long-term follow up. Increased leisure time activity did hinder this regression. With an eye on determining the optimal frequency of intervention, which achieves clinically relevant results and promotes lifelong adherence, it might be of interest to examine if any improvements could be obtained after only a single session of aquatic therapy.

Therefore, the aim of this exploratory study was to measure the immediate effects of a single session of aquatic therapy on balance, strength, and functional reach in persons with chronic osteoarthritis, older than 55 years of age. This cut-off age is based on the European Innovation Partnership on Active and Healthy Ageing (EIP-AHA), who consider persons from 55 years and older in their studies of fall risk. Martins et al. (2015) [20] showed this relation. On the other hand, Linaker and Walker-Bone (2015) presented an overview of upper limb dysfunction related with occupation and daily life activities and also found this age group to be of increased risk [21]. Our hypothesis was that functional test results should show an effective significant change after one aquatic therapy session.

## 2. Materials and Methods

### 2.1. Study Design and Setting

A quasi-experimental trial was carried out, with participants recruited from the Hospital after ethical approval. First, the volunteers filled in a sociodemographic data and health condition questionnaire, the disability arm shoulder and hand (DASH) scale and signed the informed consent. An expert physiotherapist performed a fixed protocol of aquatic therapeutic exercises each session. Three physiotherapists performed the measures: Stadiometer scale, balance weighing scale, visual analogic scale for pain (VAS_Pain), step test (ST), functional reach test (FRT), and the global balance standing test (GBST), before and no more than 2 min after the intervention. The control group without aquatic therapy intervention followed the same procedure, with measurements taken before and after 45 min of a rest period in a chair. The study design and reporting follow the CONSORT recommendations for conduction and reporting. This was not a randomized controlled trial [22].

### 2.2. Subjects

**Figure 1:**
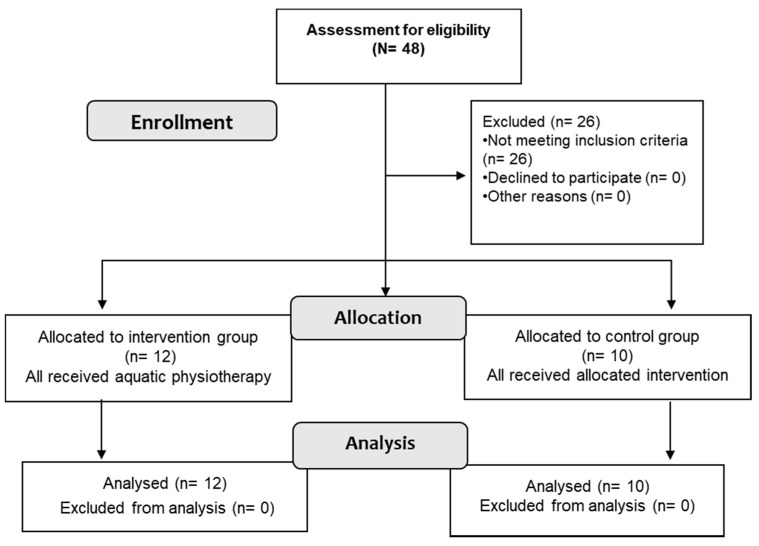
Study design (CONSORT 2010).

From the hospital falls risk assessment program, all potential subjects 55 years and older were invited to participate. A sample of 48 subjects was available for eligibility assessment. Twenty-two participants met the inclusion criteria and agreed to participate in the study. All participants provided informed consent according to the Helsinki Declaration and Oviedo Convention. All 22 participants experienced one or more of the following: Disability of upper limb, problems with balance and/or lower limb weakness. The stratified allocation resulted in an experimental group with poorer health. In practice, those persons had an indication for aquatic physiotherapy to decrease body impact. The control group was of better health and took part in land activities during therapy and had less body impact problems. The intervention group (*n* = 12) included those with several health problems on the waiting list to start an aquatic therapy program. These persons had some previous experience in this type of therapy but were coming from a wash-out period during the 2 months summer vacation break prior to the study. The remaining sample with upper limb dysfunction, fewer health problems, and little or no experience with aquatic therapy were waiting for a fall risk assessment as part of a general functional health screening. These were included in the control group (*n* = 10), as healthier older persons but with upper limb disability and balance problems (Figure 1). It was essential that the experimental group have at least some experience in aquatic therapy to assure that the single session could be organised efficiently. The inclusion criteria were: (a) No severe mobility deficits, (b) be able to walk and stand independently, and (c) do not present mental disorders or deficits in communication and understanding instructions. Individuals with severe mobility deficits (*n* = 14), inability to walk or stand independently (*n* = 6), and individuals unable to communicate or understand commands (*n* = 6) to perform the proposed activities were excluded.

### 2.3. Procedures for Data Collection

Initially, when volunteers agreed to participate in the study, they completed a written questionnaire for sociodemographic data, health conditions, informed consent, and DASH scale. The data for the intervention group were collected in the pool building in a quiet room, temperature 25 °C, with the participants wearing comfortable clothes (t-shirt and swim suit). We started by measuring the height and body mass using a stadiometer and body weighting scales. The outcomes measurements (VAS_Pain, ST, FRT, GBST) were then made immediately before and after the 45 min aquatic therapy program (not more than 2 min after the session of aquatic exercises). To decrease the bias, researchers used the same protocol [23] in three sessions to collect data of four participants in each session. The data collection of the control group took part in the hospital sport hall. First, all participants completed the written questionnaire for sociodemographic data, health conditions, and DASH scale, and signed the informed consent. After this, the outcome measurements (VAS_Pain, ST, FRT, and GBST) were taken before and after sitting quietly for 45 min without the activity.

### 2.4. Intervention

The aquatic therapy intervention used a protocol with upright exercises (walking in different patterns and directions, lower limbs movements, sitting and standing) [24] and active relaxation exercises from the Halliwick and Clinical Ai chi methods [18,25]. Each session lasted 45 min and included a warm-up, conditioning, and cooling down period. The warm-up included gait exercises in all directions, with a change of pace, gait with dissociation of the waist, and walking on toes and heels [7,23]. The main objective of conditioning was to improve balance. Exercises such as bicycling with legs, upper limb reach, pushing the water, and slips with a noodle float support were included. Balancing exercises were also performed in the sitting and standing positions with floating plates [8,26,27] and Ai-chi movements with exercises in unipodal or bipodal support according to the participant’s tolerance [18,28]. Cooling down included gait exercises with waist dissociation, standing-balancing with Ai-chi upper limb movements (first five movements), followed by relaxing cervical movements, shoulder rotations, and slow stretches [29,30,31,32,33,34].

### 2.5. Outcomes for Data Collection

The DASH is a scale that assesses the perception of upper limb functionality. It consists of 21 questions related to pain or other symptoms, activities of daily living, leisure activities, and professional activities. A lower score indicates a musculoskeletal upper limb problem and disability [35]. VAS_Pain is a 10 cm scale where the users quantified their pain from 0 to 10 in real time in a sheet of paper with the scale printed. Zero is no pain and 10 is considered the worst pain imaginable. The score is categorized as follows: Less than 3 cm—little pain, between 3 and 6.9 cm—moderate pain, equal to or more than 7 cm—severe pain [36], with a minimal clinically important difference found for adults with musculoskeletal as 1.4 cm change [36,37,38].

**Figure 2:**
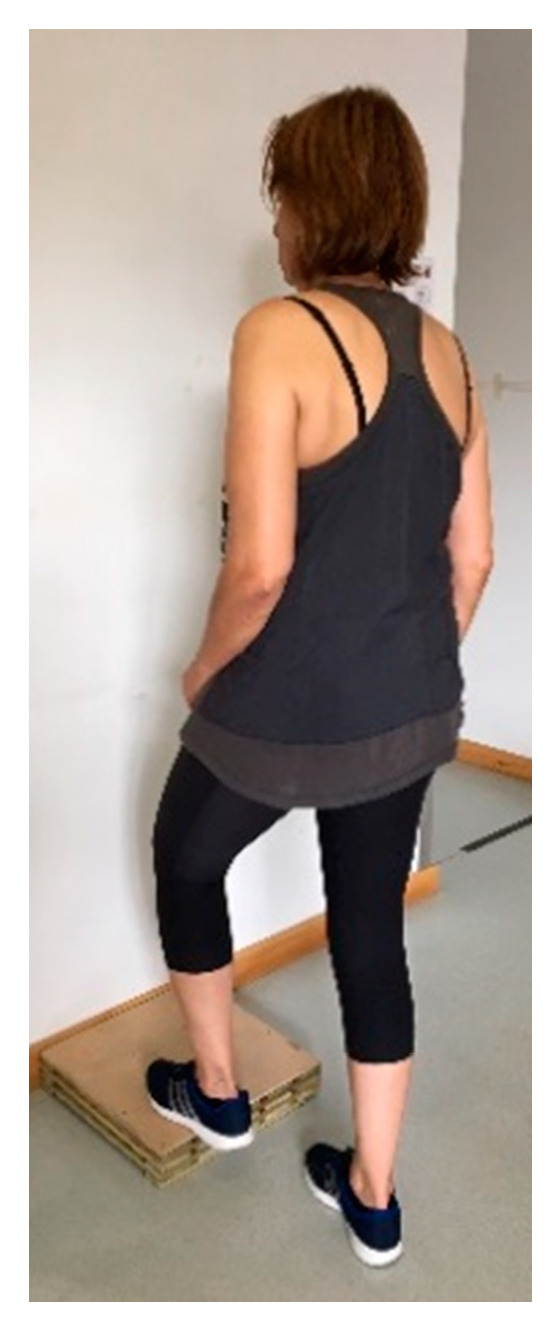
Step test example.

The ST is a test of dynamic balance. The users were instructed to stand in front of a step 7.5 cm high without support while simulating the ascent and descent of stairs alternately on the right and left foot (Figure 2). Complete repetitions were counted during 15 s [39]. The mean values of three trials were recorded. The speed of performance in the step movement provided a significant prediction of non-fallers with a success rate of 70%. Seven or less steps in 15 s suggested high instability [15].

**Figure 3:**
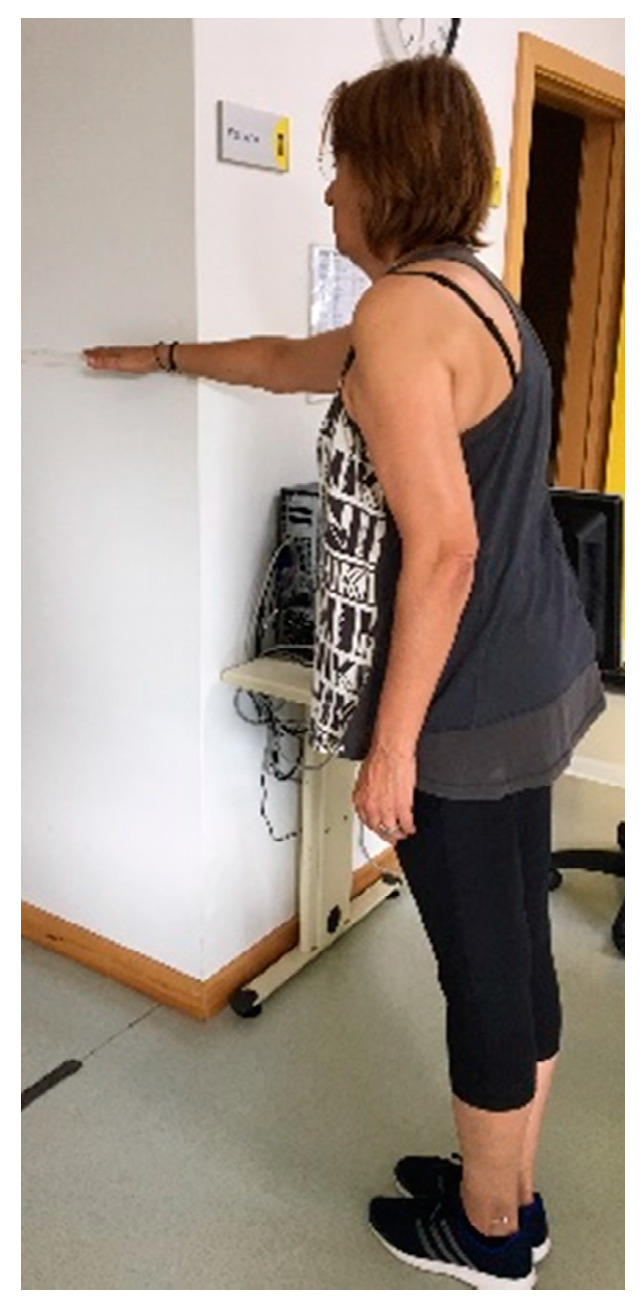
Functional reach test example [41].

The FRT is a test that evaluates the frontal dynamic balance. The patient must be able to stand independently for at least 30 s without support and also be able to flex the shoulder to at least 90° (Figure 3). The participant was instructed to stand next to a wall so that he can reach forward along the length of the metric stick as far as possible. The movement is repeated three times and the evaluator measures the distance achieved in centimeters [40]. The mean of the second and third attempt is recorded. The performance in FRT should be between 15 and 25 cm to be considered as a safe frontal dynamic balance [41].

The GBST on the force platform (Hercules model from Sensing Future, 60 × 48 × 45 cm), used Wi-Fi to monitor communication, acquisition frequency until 100 Hz, and software (from Sensing Future) to evaluate the static balance with biofeedback in the computer monitor, using a red and green cross as an indicator of bad or good equilibrium, respectively [42]. The equation for calculating the percentage in equilibrium is as follows:

(1) $$\%balance=\left(\frac{equilibrium\;time\left(in\;seconds\right)}{total\;time\;\left(in\;seconds\right)}\right)\times 100$$

Equation (1)—Calculating percent values of equilibrium time on the force platform.

**Figure 4:**
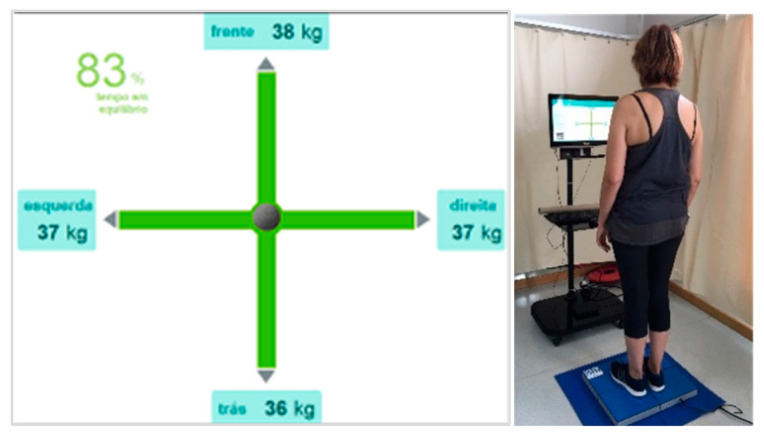
Monitor picture of physio sensing platform for feedback on balance.

The equilibrium time is the time inside the green indicator. In other words, when the weight difference between the left and right side and the difference of weight between the front and back are within the defined tolerance. Tolerance (5%) is the allowable weight margin for the indicator to remain green (Figure 4). Each user was asked to stand up straight and completely still, with eyes open, during 1 min and repeat it three times. The best score is recorded, in trials for which at least 70% of the time in the equilibrium zone was achieved with biofeedback to have safe equilibrium reactions [20,43].

### 2.6. Ethical Procedures

Researchers received permission from the local hospital Ethics Committee (07/12/2017-CE) and National Data Protection Commission (number 7103/2017) to treat the data and publish the results.

### 2.7. Statistical Analysis

The analysis was conducted with the statistic software SPSS version 24.0 (SPSS Inc., Chicago, IL, USA). Twelve participants were allotted to the intervention group as recommended by Julious et al. (2005) for a pilot study [44]. The normality of all outcome variables was assessed using the Shapiro-Wilk test and homogeneity of variances were verified using the Levene test. Two-way repeated-measures ANOVA allowed comparing data before and after the intervention and between groups (experimental and control) for VAS_Pain, ST, FRT, and GBST. For all ANOVAs, Mauchly’s sphericity test was performed, and where this assumption was violated, Greenhouse Geisser adjustments were used [45,46].

## 3. Results

After the baseline assessment, all participants were included in the data analysis. Subsequently, 22 participants, 12 in the intervention group and 10 controls took part. No outcome measures showed any significant deviation from the normal distribution. No differences were found regarding age, height, weight, and BMI between groups, but there are differences for gender, health conditions, medication, osteoarthritis status, and DASH. The proportion of subjects with health conditions, medication, and osteoarthritis was higher in the intervention group. Although all participants had experienced some upper limb disability, at the time of data collection the control group had a lower DASH score (Table 1).

Results showed better values after the aquatic intervention for the VAS_pain (6.2 (1.9) vs. 5.2 (2.3)) and for the GSBT (61.7 (5.9) vs. 71.3 (18.2). The analysis showed a time effect but no significant changes in the intervention group. There are no differences between groups in the post-test (ANOVA *p* < 0.05) results but the control group was better in the pre-test (*p* < 0.01). Actually, immediately after the intervention, the scores of all tests increased in the intervention group but remained almost unchanged in the control group (Table 2).

## 4. Discussion

The purpose of this exploratory study was to evaluate the immediate effects of one aquatic therapy session in a patient’s group older than 55 years, following a wash-out period of no aquatic therapy after the summer vacation. Many studies look for improvements due to longer interventions [6,8,25,30,33,36,37], but in this study, the key point was to find what changes occur immediately after a single training session. The literature showed significant benefits in several patient groups [47], however, the outcome measures used in previous studies were not consistent. Due to the limited time for assessment, researchers chose measures relevant to the health problems of the participants, as well as valid, reliable, and sensitive to training [6,17,38,39,40].

In the current study for pain evaluation, the VAS_Pain showed a 1 cm improvement in the intervention group, while the control group showed no change after 45 min of sitting rest. No minimal clinically differences were shown in accordance with Salaffi et al. (2004), who proposed 1.4 cm as the minimal clinically important change [38]. On the other hand, Simmerman et al. (2011) found efficacy in two sessions of aquatic vertical traction in patients with persistent low back symptoms with changes in spinal length and pain, 2.7 (2.1) cm. As in our study of one session, pain deceased [48]. For the step test, the intervention group showed a score of about seven steps both before vs. after the intervention. These results might be affected by poor one-foot standing balance in the intervention group. The control group had better baseline results of about 11 steps, with no change before vs. after [40,49].

The literature suggests functional reach test values between 25.5 and 28.9 cm as an indication of good functional reach test performance in community-dwelling older adults [4,41]. Our intervention group results were more than 15 cm under this level and did not change with a single session of aquatic therapy. The control group showed better scores (≈20 cm) suggesting a moderate balance control. There were no minimum important changes, which suggested no improvement with the aquatic physiotherapy, but also no effect on the test learning process in the control group [50].

Finally, for the global balance standing test [5,18,40] based on the predictive study of Martins et al. (2015), a person should be more than 60% of the time within balance in the test to predict a low risk of falling. The results showed improved performance in the GBST for the intervention group, whereas this change was not observed in the control group. However, there was no significant interaction as the intervention group had a significantly lower baseline performance than the control group. The improvement, although not significant, did bring the intervention group up to the level of the control group. This suggested some influence of aquatic therapy in the standing balance for older adults with upper limb disability and that they do not loose balance even shortly after an aquatic session.

On the other hand, the control group showed better baseline results compared to the intervention group. Our control group were a non-active sample to study the influence of the repetition (learning effect) on the functional measurements. However, even with a better baseline, we expected some effect of learning and/or resting [51]. In addition, as the control group had lower values on BMI, with higher height, lower weight, and less additional health problems, we might have expected more and greater baseline differences.

The intervention group showed a tendency toward greater improvements in the outcomes used particularly in the global balance test, although no significance was found. The immediate changes of aquatic therapy observed in this sample, reinforces the importance of patients with upper limb dysfunction repeating a series of aquatic therapy sessions annually although the immediate improvement was not significant. As function decreases with age, we recommend aquatic therapy programs for maintenance of function as age increases [52,53]. Furthermore, the study of Cronin et al. [54] suggested that water immersion may offer promise as a low-risk, non-invasive, and non-pharmaceutical method of decreasing peripherally reflex excitability in persons with hemiplegia. Therefore, relaxation can be related with a decrease of peripheral reflex excitability.

The limitations of this study are related to the non-randomization, the different baseline level of the groups, and the study of only a single aquatic therapy session. However, this exploratory study pointed out no differences. On the other hand, the results of the experimental group showed a slight decrease in pain and an increase in balance with one single aquatic therapy session. Importantly, there were no decrements in performance. Moreover, the current study suggests that this population with fragile health (11 vs. 1) and osteoarthritis (10 vs. 2) needs to remain active (in aquatic therapy) and our immediate effect results re-enforce this goal.

Further studies should measure one single session several times to understand the evolution of the participants in a long-term program. Further, the monitoring of physiological and kinematic parameters, related to the clinically relevant expectations, can be important to better understand why returning patients are attracted to aquatic therapy in long-term programs. The establishment of the minimal important clinical changes could provide important goals and motivation for patients and physiotherapists in the practice performance.

## 5. Conclusions

This study had no immediate functional effects. No decrements in performance on balance or pain were found. Findings showed that participants in the intervention group experienced immediate positive changes in one single session, which enforces the motivation to continue the aquatic therapy program. The outcomes suggest that people older than 55 years have a disability in the balance test and risk. For future studies, the measurement of one single session several times is needed to understand the participant’s evolution in the long-term program. Additionally, an important goal will be to establish a minimal observable difference for one single session of an aquatic therapy program for persons older than 55 years with upper limb dysfunction and other related problems of this age group.

## Figures and Tables

**Table 1:** Baseline characteristics of the participants (mean/standard deviation (SD)).

Characteristics	Intervention		Control	
	Female	Male	Female	Male
Gender/n	11	1	7	3
Age (years)/mean (SD)	62.9 (5.9)	58	61.9 (5.6)	75 (8.7)
Height (m)/mean (SD)	1.6 (0.1)	1.7	1.7 (0.8)	1.7 (0.7)
Weight (Kg)/mean (SD)	70.6 (12.4)	100	65.9 (11.2)	72.0 (11.8)
BMI (kg/m^2^)/mean (SD)	27.8 (4.1)	35	27.0 (4)	25.0 (2)
Health condition * (yes/no)/n	11/0	1/0	4/3	1/2
Medication (yes/no)/n	7/4	0/1	1/6	0/3
Osteoarthritis (yes/no)/n	10/1	1/0	1/6	0/3
DASH Score/mean (SD)	54 (13.8)	67	28.7 (21.8)	6.11 (5.9)

* (High blood pressure or high cholesterol or diabetes mellitus 2).

**Table 2:** Changes due to the immediate effects on pain and balance performance after the aquatic therapy, two-way repeated measures ANOVA.

Outcomes		Before Mean (SD)	After Mean (SD)		
VAS_Pain (cm)	Intervention (*n* = 12)	6.2 (1.9)	5.2 (2.3)	Time: F (1.20) = 4.31	*p <* 0.05
				Time*group: F (1.20) = 3.86	*p* = 0.06
	Control (*n* = 10)	0.9 (1.9)	0.9 (1.9)	Group*inter subjects: F (1.20) = 97.60	*p <* 0.01
ST (n)	Intervention (*n* = 12)	7.0 (2.0)	7.4 (1.8)	Time: F (1.20) = 11.20	*p* < 0.01
				Time*group: F (1.20) = 0.39	*p* = 0.54
	Control (*n* = 10)	11.23 (3.1)	11.80 (3.3)	Group*inter subjects: F (1.20) = 297.04	*p* < 0.01
FRT (cm)	Intervention (*n* = 12)	9.1 (2.8)	10.4 (3.8)	Time: F (1.20) = 4.67	*p <* 0.05
				Time*group: F (1.20) = 1.31	*p =* 0.27
	Control (*n* = 10)	20.41 (5.9)	20.91 (5.5)	Group*inter subjects: F (1.20) = 270.43	*p* < 0.01
GBST (% T in balance)	Intervention (*n* = 12)	61.7 (5.9)	71.3 (18.2)	Time: F (1.20) = 2.24	*p* = 0.15
				Time*group: F (1.20) = 3.34	*p* = 0.08
	Control (*n* = 10)	73.24 (24.3)	72.24 (22.9)	Group*inter subjects: F (1.20) = 237.80	*p* < 0.01
